# The plant detectives: innovative undergraduate teaching to inspire the next generation of plant biologists

**DOI:** 10.3389/fpls.2015.00729

**Published:** 2015-09-15

**Authors:** Elizabeth A. Beckmann, Gonzalo M. Estavillo, Ulrike Mathesius, Michael A. Djordjevic, Adrienne B. Nicotra

**Affiliations:** ^1^Centre for Higher Education, Learning and Teaching, Australian National University, ActonACT, Australia; ^2^CSIRO Agriculture, Crop Adaptation, ActonACT, Australia; ^3^Research School of Biology, Australian National University, ActonACT, Australia

**Keywords:** undergraduate teaching, research-led education, plant science teaching, active learning, Arabidopsis

## Abstract

Encouraging more students to embrace plant science research is a global priority. We have evolved a second year undergraduate course from a standard lecture/practical format into an innovative research-led learning design that gives students hands-on experience of cutting-edge plant science research and specialist instrumentation. By making tangible the links between plant genetics, biochemistry, physiology and function, the active learning curriculum extends students to their limits, and gives them insights into the multi-faceted nature of plant science research. Using genetically-mapped mutants of *Arabidopsis thaliana*, we challenge our students to apply their conceptual learning immediately to identify “unknown” genetic mutations affecting plant form and function. By exposing students early in their student careers to the challenges, rigors and excitement of plant science research, we have helped them grow quickly into astute researchers who truly deserve the title “Plant Detectives.” Many have become motivated to continue their studies as plant biologists in research-focused honors (pre-doctoral) and doctoral programs.

## Introduction

Global food security is a daunting prospect. Faced with increasingly scarce arable land, projected population increases, and climate change (World Hunger Education Service, 2012; Charles et al., 2014), tomorrow's plant biologists will need cutting-edge techniques and technologies to harness plant morphology, physiology, and genetics to create crops with higher yields and resilience in diverse environments (Jones, 2014). These are exciting research challenges for bright undergraduate minds, but perhaps not always as immediately attractive as research on artificial intelligence or cancer. Encouraging more students to embrace plant science is thus urgent, but not always easy (Leversley et al., 2012; Australian Council for International Agricultural Research, 2014; Ebert-May and Holt, 2014; Jones, 2014). How might those of us teaching future scientists provide them with the intellectual impetus to pursue plant science?

Since, the Boyer Commission on Educating Undergraduates in the Research University (1998) reported the failure of universities to develop an adequately research-literate citizenry, there has been much discussion about research-led education (e.g., Healey, 2005; Brew, 2012). Educational theory suggests that traditional undergraduate science teaching comprising weekly lectures and laboratory sessions encourages a shallow approach to learning (Ramsden, 1992; Marton et al., 1997; Waldrop, 2015): students submit standard lab reports and rote learn to pass exams. Empirical research suggests that active learning provides much better outcomes in knowledge, understanding and application than traditional science teaching approaches (Crouch and Mazur, 2001; DeHann, 2005; Watkins and Mazur, 2013; Freeman et al., 2014; Waldrop, 2015). In this paper, we report on the evidence-based educational design features progressively incorporated into a second year undergraduate course to create a curriculum that lets students “experience learning through, and about, research and inquiry…in ways…that closely mirror the academic research experience” (Healey and Jenkins, 2009, p. 3). To capture students' curiosity about the genetic basis of plant phenotypes and physiological responses to environmental stressors, we focused on integrating laboratory investigations of plant mutants that reflect the puzzle-solving realities of research—iterative sequences of observation, hypothesis formulation, and evidence collection. We describe the highly successful educational outcomes we achieved by giving enthusiastic but inexperienced science students a realistic taste of the joys and frustrations of research, and how we are thus nurturing the new generation of much-needed plant scientists (Bradforth et al., 2015).

## Methods: using evidence-based course design

### Course structure

The course, *Plants: Genes to Environment*, helps students understand how genes affect plant form, function, and performance in the context of diverse abiotic environmental factors. By 2008, the course had already run successfully (i.e., with very good pass rates and student evaluations) for several years, but relatively few students continued studying plant science. The course originally followed a traditional Australian model: one semester (13 weeks) of three lectures, one tutorial, and one laboratory practical per week. In 2008, anticipating the subsequent “call to action” for change in undergraduate biology education (Brewer and Smith, 2009), we introduced major innovations. Still within the 13-week timeframe, we re-structured the course into three 1-h lectures and a 1-h tutorial each week for 10 weeks, and eight 3-h practicals (Supplemental Table 1). Consistent with the educational concepts of constructive alignment—whereby learning activities and assessment tasks are directly aligned with the intended learning outcomes (Biggs, 1996; Biggs and Tang, 2011)—and authentic learning (Herrington and Herrington, 2006), we focused on developing students' critical thinking and research competencies. Aware of concerns associated with “minimal guidance” teaching (Kirschner et al., 2006), we ensured effective guided instruction throughout.

### Lectures

In lectures, we introduced a structured interactive “read-think-discuss-listen-review” format requiring students to engage intensely with three sources: textbooks and online resources, their peers, and the teaching team (Taiz et al., 2015). Influenced by the peer instruction model (Mazur, 1997; Crouch and Mazur, 2001), we required students to answer specific focus questions as they read in preparation for each lecture (Supplemental Table 1). This made more effective use of limited lecturer time, and transformed lecture monologs into group learning experiences. As each course progressed, all lecturers noted that the quality of students' oral responses improved markedly: students showed increasing confidence in questioning their conceptual understanding, with the lecturer and with one another. Weekly tutorials enriched students' generic skill sets, through “how to” sessions on data analysis, research writing, bibliographic software, and gas exchange instrumentation.

### Laboratory research activities

After several weeks of in-depth theory, students form teams of two to four “Plant Detectives.” Each team is given seeds from both wild type *Arabidopsis thaliana* and an unidentified mutant (any one of 12 Arabidopsis lines harboring a point mutation in specific genes), which they must then identify using their newly-acquired theoretical knowledge and pertinent laboratory techniques. Arabidopsis is perfect in this role because of its short life cycle (6 weeks from germination to mature seed), relatively small and fully mapped genome, and non-transgenic mutants suitable for class use (readily purchased from the Arabidopsis Biological Resource Centre, USA; https://abrc.osu.edu/). The highly contrasted features of each mutant highlight genetic impacts: by comparing homozygous mutants to wild type plants, students can identify morphological or physiological differences in specific traits (Supplemental Figure 1). Detailed observations of these changes in response to environmental factors provide “clues” about the role and nature of particular genes, allowing students to solve the “mystery” of the mutation. Through a coherent set of experiments (estimation of seed germination rates; measurement of root development and gravitropism; visual examination of external/internal anatomy and morphology; pigment composition analysis; and gas exchange studies), students apply their lecture-gained knowledge to the puzzle of “identifying the mutation” through comparisons with published literature and databases. As only the Lab Co-ordinator knows which mutants are assigned to which team (and hence, which tests may prove useful in identification), both students and teachers share the “mystery.” Reflecting the nature of authentic research, shared ignorance leads to a shared goal and a lively inquiry process—part of our deliberate strategy to spark students' natural curiosity to motivate them to invest both their time and their intellectual effort into their learning.

However, this kind of experiential learning work is high-stake: we risk demoralizing students if they perceive workloads as greater than more traditional courses, or if too many experiments have “negative” results. We therefore incorporated design features to limit potential student frustration or anxiety. First, although we wanted students to learn that negative results are as important in research as positive ones, we selected mutant lines that would respond differently to wild type plants in at least one of the eleven research activities, thus ensuring that no team had solely negative outcomes in their experiments. Recognizing students' limited experience in laboratory techniques, we developed a Manual—refined regularly through student input—with step-by-step protocols, safety advice, technical preparation information, and guidelines for data analyses, report-writing, and oral presentation (Supplemental Table 2; Estavillo et al., 2014). Consistent, compulsory pre-practical online activities encourage students to read the Manual before labs, and help students learn how to develop investigative hypotheses before embarking on experiments.

In the lab itself, students are supported by one or more lecturers, an experienced demonstrator, a technical officer, and—since 2010—one or more paid Peer Mentors (enthusiastic former Plant Detective students identified through “expressions of interest”). We enhance cross-team collaboration by instituting “lab meetings” at the start of each lab session. In these small groups containing one member from each team, each student shares his or her group's results from the previous session, and compares hypotheses and objectives for the current session. These lab meetings give the students practice in communicating results, reflecting on discoveries, and taking new ideas back to their teams, hopefully mirroring the collaboration of plant science researchers across the world.

### Assessment

Assessment is always a key student concern, especially when they see a potentially “risky” emphasis on collaborative learning. We assess students' individual achievements in this course in several ways. First, in the lecture sessions, students are awarded individual pass/fail marks for engagement and interaction, specifically for (i) preparing written notes on pre-lecture study questions and (ii) presenting the outcomes of small group discussions to the whole class (at least twice each semester). Bonus marks (plus/minus 5%) reward additional, or particularly useful, contributions. Pre-practical quizzes account for another 10%. The key assessment task (50%) is a theory exam, based predominantly on the already-discussed pre-lecture study questions. The course concludes with a symposium, in which each group presents its findings (10%), before each student submits an individual paper (in the style of *Functional Plant Biology*) on the characterization of the team's mutant (30%). Students are assessed on communication, description of inquiry processes, and data presentation/analysis, but *not* on whether they correctly identify their plant's mutation: students consistently report their appreciation of our emphasis on the process of discovery and sharing of results, rather than on the “right answer.” Total marks are normalized to 100%.

## Methods: evaluating outcomes

Giving students increased responsibility for their own learning meant that we needed to listen to their feedback about that learning, so we engaged students fully in formative evaluation of the course structure. Given the relatively small enrolments (20–35), we focused on qualitative feedback. Using a design-based research model (Sandoval and Bell, 2004), approved by an ANU Human Research Ethics Protocol, since 2008 we have collected student feedback during and after each course, through anonymous minute papers and university-standardized course evaluations (2008–2014), focus groups and in-depth interviews (2009 and 2010), and Peer Mentor feedback (2010–2012). In 2014, we contacted students who had taken the course 2 to 4 years previously, and asked for their recollections of the course, and its influence (if any) on their subsequent studies or employment.

## Results

The ongoing evaluation enabled a continuous improvement cycle (Bessant and Francis, 1999) that survived changes of course convenor, practical co-ordinators, tutors, and timetabling. Students reported that the course fostered intense problem-solving and creativity as they searched for clues about the mutants:

“The different style to other courses was …refreshing. The experimental work was particularly stimulating and challenging. It gave me a glimpse into what real research would be like and a feel for some of the practicalities and difficulties” (anonymous student feedback).

We saw students negotiating with team members, design their own investigative pathways, and assess their results comprehensively and critically:

“[The course] taught me self reliance and discipline …to go into the lab every day (or every second/third day if you managed these duties well as a team) to measure seedling growth, note the direction of growth …so you had solid data with which to form your clues. …you taught us the skills we needed to complete the task” (student feedback, 2014, 2–4 years post-course).

**Figure 1:**
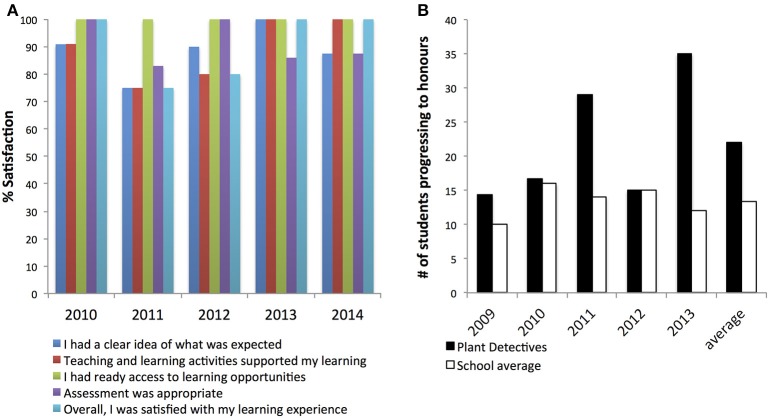
**(A)** University-standardized course evaluations by students 2010–14, **(B)** Proportion of students progressing to Honors (Plant Detectives vs. all Biology School). There was a change of course convenor in 2011.

In the university-standardized course evaluations, students consistently reported high levels of satisfaction across many parameters of teaching and learning (Figure 1A), including clear expectations (mean 88%), effective learning activities (mean 85%), ready access to learning opportunities (mean 100%), appropriate assessment (mean 90%), and overall satisfaction (mean 90%; 100% in 2010, 2013, and 2014 respectively).

The interactive lecturing and the pre-lecture focus questions (somewhat similar to the “flipped classroom” approach) have been very successful in encouraging engagement:

“I remember the unique style of lectures with reading beforehand and questions to be answered that fitted really well with the final assessment” (student feedback, 2015, collected 2–4 years post-course).

The students quickly saw the benefits of enhanced communication skills:

“It was really hard and challenging for me at the beginning to speak out during lectures and to have confidence in myself … but as time passed … my attitude toward learning (and life) changed for the better” (anonymous student feedback, 2011).

As each semester progressed, we found “response duties” were more often sought by weaker students (unheard of in other courses), who strategically negotiated with their peers to contribute more often, and thus gain bonus marks.

One key design change instigated by feedback related to the timing of the examination. Initially, students had a full semester before having to demonstrate exam mastery of theory, but student feedback encouraged us to compact the theory elements and make the examination earlier. This motivated students to maximize their learning of theory before and during their lab work:

“[Despite] having not studied plants in any depth previously, I was still quickly able to get up to speed …I found myself interested enough to start reading …outside of study hours” (student feedback 2014, 2–4 years post-course).

So effective was this shift in exam timing that overall student achievement was highest in cohorts with earlier scheduled exams. Even though, its content has evolved, the theory exam provides a valuable benchmark of student learning over time. (As this is a continuing course, the actual marks, means, or spread of marks, cannot be shared here for university and legislated privacy reasons.)

The improvements in students' theoretical learning is matched by the impact on students' technical research skills:

“I learned more from the labs in this course than all the other labs from my other courses combined” (anonymous student feedback).

Many students commented on the authenticity of the experimental work:

“The lab, while long and complicated, was an excellent introduction to longer term research objectives. … It felt more like actually doing science than playing at it”;

“The labs actually felt like doing real “science,” and were definitely more satisfying than other lab practicals I have done” (anonymous student feedback).

The realistic approach to communicating and sharing results was also valued:

“[The symposium was] a surprisingly relaxed exchange of ideas … the [staff] were genuinely interested … seeing all the other groups' work helped me think about what my group had done in clearer terms” (extract from student's public internet blog).

The Peer Mentors worked closely with the student teams to bridge the gap between researcher and student. In 2013, one Peer Mentor evaluated the peer mentors' role as a for-credit research project, developed a Peer Mentor's Handbook, and showed how the Peer Mentors also gained as learners:

“Being a peer mentor was an exciting adventure … [helped me] sharpen my communication skills and refresh my knowledge of plant science” (feedback, peer mentor, 2010).

The very high quality of students' learning—linking mutations to plant biochemistry, function, form and performance, and thinking like a researcher—is powerfully expressed in this retrospective feedback (from a student who became a Peer Mentor and went on to doctoral research in plant science):

“Apart from changing my perspective on teaching, I gained a new appreciation for the scientific method …The mutant plants my group were given to study had a [Name A] mutation, but I was convinced it was a [Name B] mutation (which can also result in severely reduced levels of [named substance], which the TLC and HPLC results supported). [The Name B] mutations also cause changes to hypocotyl length, and this was not apparent in the gel-grown plants—but I still wanted to believe I'd found the right mutant. When I discovered it was a [Name A] mutant, I realized that I wasn't following the scientific method or being generally rational toward the reverse genetics process. … this experience will help me be more scientific and rational” (2014, 2–4 years post-course).

At an Australian research-intensive university, an important indicator of a course's influence and success is the progression of its students into Honors (pre-doctoral) research programs. Usually, about 5% of all Biology students continue into Honors. Even though our students are not from a selective research-focused cohort, this course has increased both the relative proportion, and absolute numbers, of students progressing to plant science Honors: 15% of the 2007 cohort; 30% in 2008; 50% in 2009; and 40% of the 2010–2013 students (Figure 1B). Feedback from students contacted 2 to 4 years after course completion shows clearly how the course had inspired them to continue studying plant science:

“Definitely, [this course] set me on the direction of plants. After seeing the breadth of topics in plant science and gaining confidence in working [with] Arabidopsis, I was pretty set on doing biology in a plants system whereas previously I think I probably would have gone down the human genetics route.”

“ … opened my eyes to how plants are different to animals. … I ended up doing a plant specialization minor, which I was not planning to do at the start of my degree …partly due to this course as it opened my eyes to how interesting plants can be.”

“… I changed my major to Plant Science after doing this course”.

## Conclusion

“… one of the most memorable courses of my whole degree.” “I loved [this course] and have already recommended it to my first-year friends.” “One of the most interesting and influential courses I have taken in my degree.” “This was the best course I have taken so far during my time at university, not only for the academic/scientific knowledge I gained, but for the invaluable lessons regarding the importance of team work and interpersonal relationships.” (Anonymous student feedback).

Like, Wieman (2004, 8–9), we believe that “a meaningful science education involves transforming the way in which students think by promoting a progression from “novice” to “expert” in both their attitudes and their approaches to the discipline and problem solving in that discipline.” We redesigned a conventional plant “structure and function” course into one that authentically models the trials, tribulations, collaborative learning, and excitement of hypothesis-driven plant science research. Our evidence-based teaching innovations have produced a cutting-edge, research-led curriculum that—as encouraged by the Boyer Commission on Educating Undergraduates in the Research University (1998), Healey (2005), and Ramsden (2008)—engages, motivates and inspires students and teachers in a collaborative, inquiry-based intellectual challenge that gives insight into the international nature and relevance of science. Our outcomes concur with those of a recent meta-analysis: students engaged in active learning science programs significantly outperform those in more traditional lecture-style programs (Freeman et al., 2014). Similarly, Watkins and Mazur (2013) found science students who have experienced active learning are more likely to stay in that discipline.

**Figure 2:**
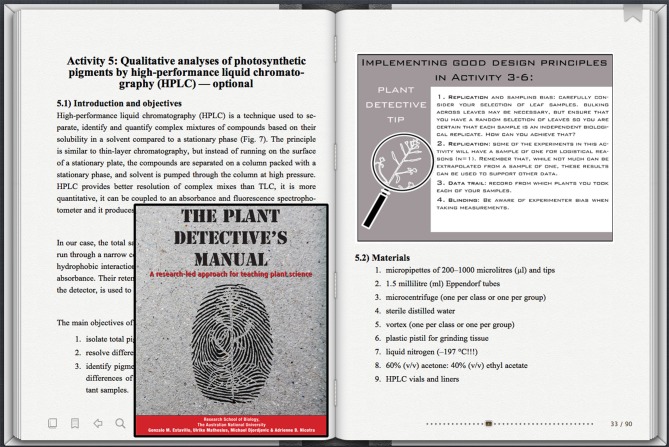
**Sample pages of Plant Detective's Manual (Estavillo et al., 2014)**.

Encouraged by multiple university and national teaching awards (e.g., Australian Learning and Teaching Council, 2011), we are keen to support other academics to attract more researchers to plant science. Following requests from national and international colleagues, the *Plant Detectives Manual* has now been published, (with animations in the ePub version, and complementary assays to suit diverse needs) and is available for free download (Figure 2; Estavillo et al., 2014). Our model is fully adaptable: the research tools and Arabidopsis mutants are readily available, and the structured-discussion fits most science teaching contexts. As researchers, we are proud to have already created the evidence-based, authentic and research-led undergraduate science teaching that has recently been advocated by Bradforth et al. (2015) and Waldrop (2015).

“Perhaps the best course that I have taken in my 3 years at the [University]. The formula is near perfect” (student feedback, 2014, 2–4 years post-course).

## Author contributions

AN convened and taught into the course from 2001–2010, UM taught in the course from 2002 to present, and GE was the practical convenor from 2008–10. MD taught into the course from 2010, and convened the course from 2011 to the present. EB was involved in educational design and evaluation from 2008, and took the primary role in writing this paper. All authors contributed to the intellectual content of the teaching and educational research, and to the drafting and revision of this paper, and fully share all accountability.

### Conflict of interest statement

The authors declare that the research was conducted in the absence of any commercial or financial relationships that could be construed as a potential conflict of interest.

## Supplementary material

Supplemental Figure 1 **Arabidopsis plants. Left:** a wild type *Arabidopsis* plant of the ecotype Columbia (Col). **Right:** an *aba2*-3 mutant, (Koornneef et al., 1982) deficient in abscisic acid (ABA)–in addition to reduced growth and size, *aba2-3* plants have much higher leaf transpiration rates and wilt faster than the wild type at low relative humidity.

Supplemental Table 1 **Syllabus of course by week and activity**.

Supplemental Table 2 **Activities included in laboratory practical sessions**.
