# Supplementary material for: The plant detectives: innovative undergraduate teaching to inspire the next generation of plant biologists
Source: Front Plant Sci. 2015 Sep 15;6:729. doi: 10.3389/fpls.2015.00729 (PMC4569745; doi:10.3389/fpls.2015.00729)

Supplemental Figure 1: Arabidopsis plants. Left: a wild type Arabidopsis plant of the ecotype Columbia (Col) . Right: an aba2-3 mutant, (Koornneef et al., 1982) deficient in abscisic acid (ABA)–in addition to reduced growth and size, aba2-3 plants have much higher leaf transpiration rates and wilt faster than the wild type at low relative humidity.

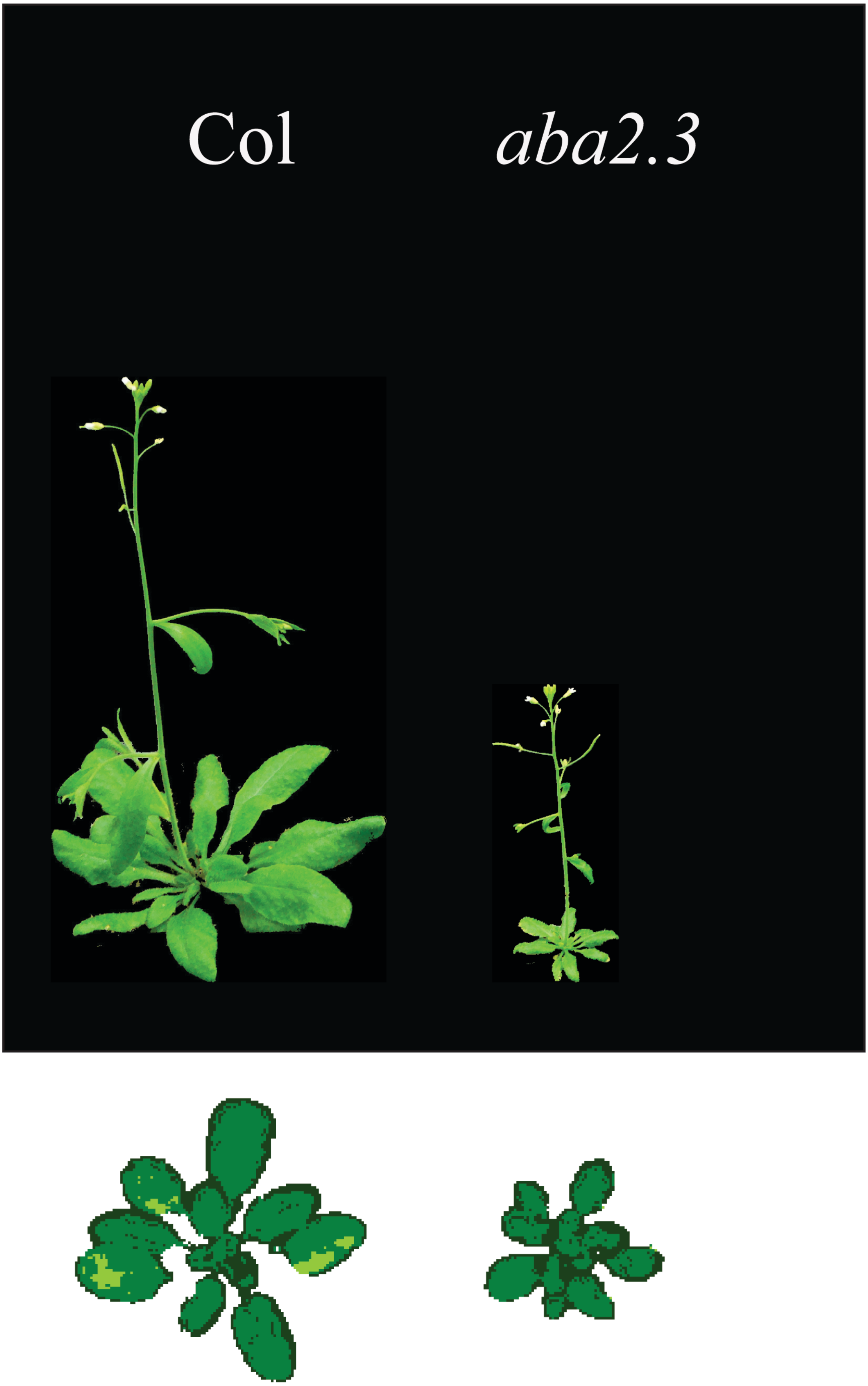

Supplement: Supplemental Figure 1 — Arabidopsis plants. Left: a wild type Arabidopsis plant of the ecotype Columbia (Col). Right: an aba2-3 mutant, (Koornneef et al., 1982) deficient in abscisic acid (ABA)–in addition to reduced growth and size, aba2-3 plants have much higher leaf transpiration rates and wilt faster than the wild type at low relative humidity. [file Image1.PDF]
