# Supplementary material for: The plant detectives: innovative undergraduate teaching to inspire the next generation of plant biologists
Source: Front Plant Sci. 2015 Sep 15;6:729. doi: 10.3389/fpls.2015.00729 (PMC4569745; doi:10.3389/fpls.2015.00729)
Supplement: Supplemental Table 1 — Syllabus of course by week and activity. [file Table1.PDF]

Beckmann, E.A., Estavillo, G.M., Mathesius, U., Djordjevic, M.A. & Nicotra, A.B. (2015) The Plant Detectives: innovative undergraduate teaching to inspire the next generation of plant biologists. *Frontiers in Plant Science,* **6**.

Supplemental Table 1. **Syllabus of course by week and activity.**

Week	Lectures	Tutorials	Laboratory Session
**1**	Inspiration: importance of plants functioning in their environment.	Explaining fundamental connection between plant genes, metabolites and phenotypes.	Independent study session
**2**	Basic understanding of how water shapes cells and how water movement into plants is determined.	Information literacy—skills for finding relevant publications in plant science.	Independent study session
**3**	Understanding nutrient trans-port and root adaptations to low nutrient availability in plants.	Critical analysis of a research paper, understanding how to critically read the introduction and frame research questions.	Independent study session
**4**	Basics of plant growth and development; control of phenotypes by plant hormones.	Critical analysis of research paper continued	Independent study session
**5**	Roles of auxin, cytokinin, abscisic acid and other regulators of plant development and drought responses.	Introduction to Plant Detectives project.	Phenotyping of Arabidopsis seed germination. Analysis of root growth in response to gravity.
**6**	Understanding function of leaves, stems and flowers.	Creating bibliography with software.	Pigment extraction and analysis by thin layer chromatography (TLC) and spectroscopy.
**7**	Understanding components of plant growth, strategies and functional traits.	Statistical analysis of scientific data – concepts of experimental design.	Pigment analysis by high pressure liquid chromatography (HPLC).
**8**	Understanding plant water relations and long distance water transport.	Independent study session	Gas exchange measurements (stressed and unstressed plants).
**9**	Detailed understanding of light and dark reactions of photosynthesis.	Computer-based statistical analysis of teams' scientific data.	Phenotyping of drought-stressed plants. Gas exchange measurements.
**10**	Review of all lecture material – Q&A and 'the big picture'	Computer-based statistical analysis of scientific data obtained in practical project.	Analysis of stomatal responses to ABA in leaf peels. Basic microscopy / histology (leaves/ stems).
**11**	Examination (on material in lectures).	Independent study session	Optional follow-up or repeat experiments
**12**	Discussion/evaluation of course.	Data analysis and discussion of results.	Symposium: Presentation of team outcomes.
**13**	Independent study session	Independent study session	Submission of research paper
