# Supplementary material for: The plant detectives: innovative undergraduate teaching to inspire the next generation of plant biologists
Source: Front Plant Sci. 2015 Sep 15;6:729. doi: 10.3389/fpls.2015.00729 (PMC4569745; doi:10.3389/fpls.2015.00729)
Supplement: Supplemental Table 2 — Activities included in laboratory practical sessions. [file Table2.PDF]

Beckmann, E.A., Estavillo, G.M., Mathesius, U., Djordjevic, M.A. & Nicotra, A.B. (2015) The Plant Detectives: innovative undergraduate teaching to inspire the next generation of plant biologists. *Frontiers in Plant Science,* **6**.

Supplemental Table 2. Activities included in laboratory practical sessions.

No	Activity title	Components
1	Observing plant phenotype	Leaf number, shape, size, morphology, pigmentation, rosette diameter Stem (bolt) height Flowering time, flower number / size, Relative Growth Rate, analyses of digital images
2	Seed germination and root growth	Germination rates, root traits (e.g. length, mass, colour, branching pattern), root growth analyses, gravitropic response
3	Extraction / quantification of photosynthetic pigments	Pigment extraction, spectrophotometric quantification
4	Qualitative analyses of pigments by thin layer chromatography (TLC)	Pigment concentration (for TLC) Thin-Layer Chromatography, pigment identification
5	Qualitative analyses of pigments by HPLC[#]	Total pigment extraction, high performance liquid chromatography analysis (HPLC)
6	Quantification of anthocyanins	Anthocyanin extraction, spectrophometric quantification
7	Gas exchange measurements on intact leaves	Light response curve, CO_2 response curve
8	Measuring stomatal conductance to water using a porometer	Porometer calibration, stomatal conductance estimation with porometer
9	Drought response	Relative water content determination, rosette dehydration experiment, gas exchange, harvest for biomass allocation, leaf areas and leaf mass per unit area determination
10	Microscopy analyses	Preparation / examination of root and stem cross-sections
11	ABA effect on stomatal behaviour	Stomatal index, ABA-induced stomatal closure
12	Write up and symposium	Manuscript write up, oral presentation
